# The relationship between the development of response inhibition and intelligence in preschool children

**DOI:** 10.3389/fpsyg.2015.00802

**Published:** 2015-06-11

**Authors:** Hon Wah Lee, Yu-Hui Lo, Kuan-Hui Li, Wen-Shin Sung, Chi-Hung Juan

**Affiliations:** ^1^Institute of Cognitive Neuroscience, National Central UniversityJhongli, Taiwan; ^2^Brain Research Center, National Central UniversityJhongli, Taiwan

**Keywords:** response inhibition, intelligence, development, age-related differences, preschool children

## Abstract

Building on the theoretical framework that intellectual behavior relies on one's ability to process both task-relevant and task-irrelevant information, this study aimed to empirically investigate the association of response inhibition with intelligence in preschool children's development. In a sample of 152 typically developing children aged between 3.6 and 6.6 years, we found evidence that suggests that inhibitory control is linked to age-related differences in intelligence. Stop-signal inhibition improved at a rate similar to the age-related changes in Verbal IQ. Components of variance analyses revealed that stop-signal reaction time predicted a larger proportion of the age-related variance in children's verbal intelligence than non-age-related variance. Results are discussed with respect to possible explanations for this intriguing relationship between response inhibition and the verbal aspects of intelligence.

## Introduction

Inhibitory efficiency is of particular developmental interest because of its close connection to learning effectiveness and appropriate social behaviors. Therefore, understanding the development of inhibitory mechanisms may shed light on changes in other aspects of cognition and behavior not only in childhood but also throughout the life span. One important but unresolved issue about the development of inhibitory control is how it is related to the development of intelligence. Research accumulated to date has pointed to seemingly conflicting conclusions about their relationship.

Inhibitory control and intelligence are sometimes viewed as two distinct, if not mutually exclusive, processes because of their different predictive validity for life outcomes. Intelligence has traditionally been shown to correlate strongly with one's educational and occupational achievement and moderately with one's social competence (Gottfredson, 1997a,b, 1998). Efficiency of self-control, on the other hand, has been found to predict not only academic achievement independent of intelligence (Bull and Scerif, 2001; Blair and Razza, 2007; Welsh et al., 2010; Chung and McBride-Chang, 2011) but also other aspects of life outcomes above and beyond intelligence, including social and emotional coping, physical health, personal finances, and criminal convictions (Carlson and Wang, 2007; Pérez-Edgar et al., 2010; Meier and Sprenger, 2011; Mischel et al., 2011; Moffitt et al., 2011; Oldehinkel et al., 2011). In addition to varying explanatory power, patient studies have also shown that these two functions are differentially impaired by frontal lobe damage. For instance, damage leading to impairment in inhibitory functions does not similarly impede performance on measures of intelligence (Stuss et al., 1983; Stuss and Benson, 1984).

However, some researchers have argued that the two constructs are in fact closely related and even proposed that inhibitory control is “a neglected dimension of intelligence” (Dempster, 1991) and “a stable component of intelligence” (Harnishfeger and Bjorklund, 1994). The theoretical basis for the relationship between inhibitory control and intelligence concerns the nature of intellectual behavior. As Dempster (1991) argues, intelligence cannot be understood without reference to the concept of inhibition because intellectual behavior relies not only on the ability to activate *task-relevant* information and processes but also on the capacity to suppress *task-irrelevant* information and processes. In this view, intelligence could be reflected in one's efficiency in handling this dual process. As such, inhibitory processes are at the core of intellectual development because better developed inhibitory control results in less intrusion of task-irrelevant information and more efficient handling of contextually appropriate information (Bjorklund and Harnishfeger, 1990). This in turn improves one's cognitive processing.

Previous studies have reported somewhat mixed findings concerning the link between the development of inhibition and intelligence. For example, several studies involving school-aged children with Attention-Deficit/Hyperactivity Disorder (ADHD) and normal controls did not find intelligence to explain inhibitory efficiency as measured by the stop-signal paradigm (Oosterlaan and Sergeant, 1996; Rubia et al., 1998; Bitsakou et al., 2008). Other studies of preschool and school children, nevertheless, reported a significant correlation between the two abilities (MacCoby et al., 1965; Loo and Wenar, 1971; Vaughn et al., 1984; Michel and Anderson, 2009). Such mixed findings could be a result of the use of age-adjusted intelligence measures. Michel and Anderson (2009) argue that the use of age norms when computing intelligence scores has essentially eliminated the effects of developmental differences; therefore, studies that used intelligence scores adjusted for chronological age were in fact assessing individual differences and thus would not be likely to find any age-related relationship between inhibitory control and intelligence. In this regard, they argue that raw IQ scores should be used when examining the developmental relationship between inhibition and intelligence. This argument seems to provide a plausible explanation for the inconsistency in the findings from the cited studies above (see Table 1 for a summary). Among the studies that used the Wechsler IQ tests, for instance, a significant relationship was found between inhibition and intelligence when raw scores were used but not when age adjustment was applied.

**Table 1:** **Summary of studies investigating the relationship between response inhibition and intelligence in children**.

**Study**	**Age range of participants**	**Inhibition measure**	**Intelligence measure**	**Relationship**
Bitsakou et al., 2008	ADHD and normal controls, 6–12 years vs. 13–17 years	Stop-signal task; Go/No-Go task; Modified Stroop task	WISC-III^a^	No
Loo and Wenar, 1971	5 years, 7 months–6 years, 5 months	Draw a Line Slowly Test; Walk Slowly Test	The Primary Mental Abilities Test (PMA)^c^	Yes
MacCoby et al., 1965	4–5 years	Draw a Line Slowly Test; Walk Slowly Test; Truck Test	The Stanford-Binet intelligence test^c^	Yes
Michel and Anderson, 2009	7–11 years	Antisaccade task	Raven's Standard Progressive Matrices^b^; The Cattell Culture Fair test^b^; WISC-III^b^	Yes
Oosterlaan and Sergeant, 1996	ADHD and normal controls, 6–12 years	Stop-signal task	WISC-R^a^	No
Rubia et al., 1998	ADHD and normal controls, 6–12 years	2 versions of the stop-signal task	WISC-R^a^	No
Vaughn et al., 1984	18–30 months	3 delay tasks (telephone task, food reward task, and gift delivery) that assess the capacity to inhibit a response to an attractive stimulus	The Gesell Developmental Schedules^c^	Yes

a Age-normed scores;

b Raw scores;

c *No indication of whether age adjustment was applied*.

To conceptualize the relationship between intelligence in development, Anderson (Anderson, 1992; Davis and Anderson, 2001) proposed a two-dimensional model in which he makes a distinction between individual differences and developmental differences in intelligence. Individual differences in intelligence refer to the variation in intellectual ability among people of similar ages, whereas age-related differences in intelligence refer to the increase of one's intellectual ability with age. Anderson argues that these two differences in intelligence should be considered as theoretically separate dimensions. According to this model, individual differences in intelligence are believed to be attributable to speed of cognitive processing, which remains largely stable across one's development, whereas age-related differences in intelligence are attributable to maturation of modules (such as inhibitory systems), which are processors that are influenced by both experience and maturationally paced factors in development (Harnishfeger and Bjorklund, 1993; Zelazo et al., 2008).

The present study was motivated by a desire to test the hypothesis that intelligence is related to inhibitory control in the context of development by using non-age-normed IQ scores. We aimed, firstly, to chart the developmental trajectories of inhibitory control and different intellectual abilities during the preschool period, and secondly, to systematically investigate how the development of inhibitory control is related to the development of intelligence in preschool children by building on Anderson's two-dimensional model of intelligence and development. On the basis of Anderson's model, it was hypothesized that inhibitory control could predict only age-related changes but not individual differences in intelligence.

Response inhibition was targeted in this study because the ability to inhibit a prepotent action is salient in children of preschool age. Previous findings have shown that it emerges at about 3.5–4 years of age (Jones et al., 2003), develops rapidly between 4 and 6 years (Tillman et al., 2008; see also Wiebe et al., 2012), and shows little development beyond ages 7–8 (Schachar and Logan, 1990). Importantly, response inhibition can be reliably measured by employing the stop-signal paradigm (Logan and Cowan, 1984), even with children as young as 4 years old (Tillman et al., 2008). The stop-signal task is particularly suited to the purpose of this study because, firstly, it can be employed to measure an individual's ability to activate task-relevant responses and suppress task-irrelevant responses. In this task, a situation is created where the participant overlearns the dominant, prepotent action (i.e., the task-appropriate go response) and, as the context changes, needs to override this ongoing action (i.e., the go response that is rendered inappropriate as a result of the stop signal). Secondly, the use of the stop-signal paradigm allows us to derive a direct measure of the participant's efficiency of inhibitory control, which is estimated based on the probability of inhibiting response to the target given a stop signal. In this paradigm, response inhibition is described in terms of a race between the go and stop processes, which are assumed to be independent and not compete for resources. The probability of response inhibition thus reflects the probability of the stop process finishing before the go process (Logan and Cowan, 1984). The stop-signal task has good validity as both an individual difference measure and an age measure of inhibitory control because a participant's stopping performance is tracked such that the probability of inhibiting is about 50% relative to one's own performance, which means that the level of difficulty is the same among participants (refer to Section Materials, Design, and Procedure for a detailed description of the design of the stop-signal task). The stop-signal task is also a relatively pure measure of response inhibition and does not seem to be contaminated by other cognitive control processes (see Verbruggen et al., 2005; Verté et al., 2006).

## Methods

### Participants

A total of 152 preschool children between the ages of 3.6 and 6.6 years (mean = 5.4 years, *SD* = 0.7 years; male = 78) participated in the study. The children were recruited from two kindergartens located in two primarily middle socioeconomic neighborhoods in northern Taiwan. All participants had normal or corrected-to-normal vision, and their age-normed IQ scores approximated a normal distribution (ranging from 73 to 135). The study was approved by the local ethical committee. Written parental consent was obtained from all the participating children prior to the study. The participants were given candy as reward upon completion of the experimental tasks. With the exception of one child who declined to complete the stop-signal task, data from a total of 151 children were analyzed. A summary of the demographic data can be found in Table 2.

**Table 2:** **Summary of demographic data and performance on the inhibition and intelligence measures**.

**Age group**	**Age range**	***n***	**% Boys**	**SSRT**		**VIQ**		**PIQ**		**RCPM**	
				***M***	***SD***	***M***	***SD***	***M***	***SD***	***M***	***SD***
4	3.63–4.25	8	75	547.78	117.83	−1.11	0.46	−0.60	0.50	7.67	0.89
4.5	4.30–4.70	24	54	467.33	101.16	−0.63	0.46	−0.94	0.59	8.00	1.17
5	4.80–5.28	33	55	438.06	111.32	−0.06	0.55	−0.18	0.47	8.00	1.66
5.5	5.30–5.79	35	43	384.58	54.85	0.40	0.35	0.13	0.49	8.57	2.04
6	5.80–6.21	35	51	356.31	59.90	0.52	0.43	0.60	0.64	10.05	2.43
6.5	6.30–6.63	15	40	322.92	29.62	0.95	0.47	0.94	0.50	13.11	1.79

*The age group labels represent the mean age of participants within an age group. SSRT, stop-signal reaction time; VIQ, Verbal IQ score; PIQ, Performance IQ score; RCPM, Raven's Colored Progressive Matrices. These are 20% trimmed means and standard deviations calculated from the Winsorized variance*.

### Materials, design, and procedure

There were three experimental tasks used in this study to measure the participants' response inhibition and intelligence: a child version of the stop-signal task, a short form of the Wechsler Preschool and Primary Scale of Intelligence-Revised (WPPSI-R), and the Raven's Colored Progressive Matrices (RCPM). The participants were tested individually in a quiet room at the kindergarten over three sessions, during which one experimental task was administered. The order of the tasks was randomized across participants.

#### The stop-signal task

**Figure 1:**
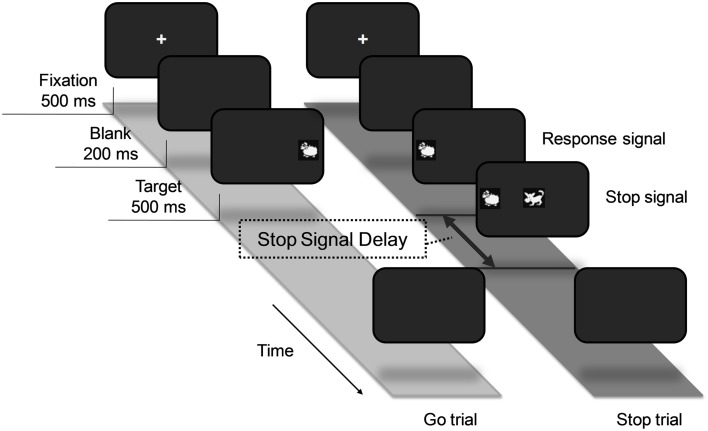
**Experimental procedure of the stop-signal task for children**. The task consisted of go and stop trials. All trials began with a central fixation cross and were followed by a sheep to either the left or right of the cross. Participants were required to press a button corresponding to the position of the location of the sheep. On 25% of the trials, a wolf would appear at the center of the screen as a signal to withhold response.

In the design of our task, a number of child-appropriate measures were used. Firstly, visual stimuli in the form of a sheep and a wolf were used as the “go” and “stop” signals respectively (see Figure 1). Each trial began with a central fixation cross which was displayed for 500 ms and then a blank screen for 200 ms, followed by a sheep to either the left or right of the fixation. Participants were instructed to press a button corresponding to the position of the location of the sheep. A stop trial would follow the same procedure described above, except that the image of a wolf would appear at the center of the screen with a delay of a pre-determined duration of time. Participants were not to press the button if a wolf appeared.

Secondly, to account for individual differences in simple RT, a block-by-block tracking method was used to obtain a participant's stop signal delay (SSD). The initial SSD was set at 170 ms after the onset of the go signal. If a participant's overall accuracy in a block was higher than 80% and half of the stop trials were successfully inhibited, the next block of trials was made more difficult by adding 40 ms to the SSD. If the criteria were not met, 40 ms was deducted from the SSD of the next block. The blocks continued until a participant was only able to inhibit approximately 50% of the responses in a stop trial, at which the estimation of stop-signal reaction time (SSRT) is found to be most reliable (Band et al., 2003). A critical SSD was obtained at this point for each participant. The critical SSD was subtracted from the mean reaction time of the correct go trials to obtain a participant's SSRT. The go RTs were filtered by removing non-response trials, trials with incorrect responses, and trials with an RT which was more than two standard deviations from a participant's mean go RT distribution.

Each experimental session began with a practice block to familiarize the participant with the task, and data from it were not analyzed. Baseline parameters for each participant were then collected by running 50 go trials to record their simple reaction time (RT) and compute the standard deviation. In a formal block, if a participant's RT in a go trial was more than two standard deviations from their simple RT, the response was considered too slow and auditory feedback in the form of a beep would be played as a reminder. A formal block consisted of a total of 32 trials, 25% of which were stop trials. The go and stop trials were randomly presented such that there was one stop trial in every four trials. A short break was scheduled after each block.

#### The Wechsler preschool and primary scale of intelligence—revised (WPPSI-R)

In this study, we used a short form of the WPPSI-R, which consisted of the Comprehension and Arithmetic subtests in the Verbal Scale, and Block Design and Picture Completion subtests in the Performance Scale. These four subtests have been found to approximate the full WPPSI-R with respect to reliability, validity, and standard error of estimate (LoBello, 1991). Because the number of items in each subtest is different, the raw scores for each subtest were transformed to z-scores and then summed respectively to create a Verbal IQ (VIQ) score and a Performance IQ (PIQ) score. The Verbal Scale measures mainly crystallized intelligence, whereas the Performance Scale measures mainly fluid intelligence.

#### Raven's colored progressive matrices (RCPM)

Participants' fluid intelligence was also measured with a computerized version of Raven's Colored Progressive Matrices (Raven, 1956). RCPM was considered appropriate for children of preschool age as it requires little verbal instruction. In this study, each participant completed 18 of the 36 items^1^ of the test (six from each of the three sets of items). To familiarize them with the format of the test, each participant was shown three similar items (one from each set) as examples before completing the test. Raw scores were used in this study.

## Results

### Preliminary analyses

Preliminary analyses revealed no gender differences in any of the measures used in this study, so gender was not included in any subsequent analyses. One participant's VIQ score was four standard deviations below the group mean and was excluded. Therefore, data from 150 participants were used in our statistical analyses. It is worthy to note that no participant in the present study experienced problems when completing the stop-signal task or had to be excluded from analysis due to poor performance. The child version of the stop-signal task that we used was able to reliably measure even emerging response inhibition between the ages of 3.5 and 4 years.

### Age-related changes in SSRT and IQ during the preschool period

**Figure 2:**
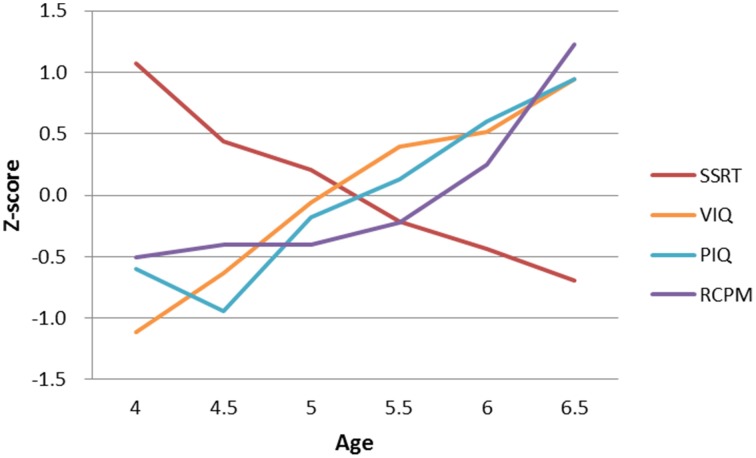
**Developmental trajectories of SSRT and different types of IQ**. Both SSRT and VIQ showed a steady progression of development between the ages of 4 and 6.5 years, but PIQ and RCPM began to develop much later, at around ages 5–6. (SSRT, stop-signal reaction time; VIQ, Verbal IQ score; PIQ, Performance IQ score; RCPM, Raven's Colored Progressive Matrices).

Participants' performance on the stop-signal task and IQ measures are presented in Table 2. To facilitate comparison across measures, Figure 2 shows the age-related changes in SSRT and the various raw IQ scores by converting to z-scores. The 20% trimmed means and standard deviations calculated from the Winsorized variances were used to chart the age-related changes because slightly non-normal distributions were observed in SSRT in Age Group 6.5, VIQ in Age Groups 5 and 5.5, and RCPM in Age Groups 4.5 and 5.5, and the SSRT distributions showed unequal variances. The use of robust estimation of location and scale using trimmed means has been shown to be less sensitive to non-normality and variance heterogeneity (Wilcox, 2012b).

We applied the robust One-Way ANOVA method using trimmed means, *t1wayv2*, and *post-hoc* pairwise comparison, *mcppb20*, suggested by Wilcox (2012a) to the data. The primary purposes of the ANOVAs were to characterize the extent to which performance improves with age and to compare the overall effect sizes observed in the different inhibition and intelligence measures. The robust measure of effect size used in this study is indicated by ξ, with ξ = 0.15, 0.35, and 0.50 corresponding to small, medium and large effect sizes, respectively (Wilcox and Tian, 2011). The use of trimmed means in analysis of variance has been found to show better Type I error protection and increased statistical power (Wilcox, 2012b).

The results showed that, as expected, older children had more efficient response inhibition and higher IQ than younger ones: SSRT, *F*_t_ = 7.10, *p* < 0.001; VIQ, *F*_*t*_ = 17.03, *p* < 0.001; PIQ, *F*_*t*_ = 11.86, *p* < 0.001; RCPM, *F*_*t*_ = 8.57, *p* < 0.001. The effect size ξ for these four measures were 0.60, 0.70, 0.70, and 0.65, respectively, indicating a very large effect of age on all measures. While stop-signal inhibition and different types of intelligence significantly improved with age in the preschool period, their rates and patterns of development differed. SSRT improved at a rate of approximately 100 ms every 12 months from age 4 to 6. A steady developmental progression was also observed in participants' VIQ in this age range, but their fluid intelligence began to develop much later, at around age 5 for PIQ and age 6 for RCPM.

### SSRT vs. individual and age-related differences in intelligence

To investigate whether SSRT is differentially related to individual and age-related differences in intelligence, correlation was first performed to test the relationships between age, SSRT and the raw IQ scores. The Kendall's tau correlation was used as slightly non-normal distributions were detected. As shown in Table 3, all correlations between SSRT and intelligence scores reached the level of significance and were negative, illustrating that participants with more efficient stop-signal inhibition showed a higher level of intelligence. However, the degree of correlations varied among the different intelligence measures. SSRT was more strongly correlated with VIQ than with PIQ and RCPM.

**Table 3:** **Correlations between age, SSRT and intelligence measures**.

	**SSRT**	**VIQ**	**PIQ**	**RCPM**
Age	0.291^***^	0.408^***^	0.391^***^	0.269^***^
SSRT	–	−0.255^***^	−0.182^***^	−0.144^**^

** Significant at 0.01 (two-tailed);

*** *Significant at 0.001 (two-tailed)*.

*Values are Kendall's tau correlation coefficients. SSRT, stop-signal reaction time; VIQ, Verbal IQ score; PIQ, Performance IQ score; RCPM, Raven's Colored Progressive Matrices*.

**Figure 3:**
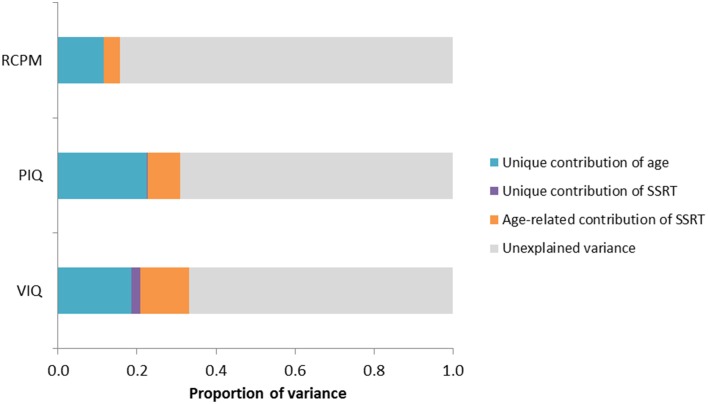
**Bar chart showing variance partitioning for VIQ, PIQ, and RCPM**. The variance explained by age was significant in all IQ scores, but SSRT significantly predicted unique variance only in VIQ but not in PIQ or RCPM. (SSRT, stop-signal reaction time; VIQ, Verbal IQ score; PIQ, Performance IQ score; RCPM, Raven's Colored Progressive Matrices).

Since SSRT and all the intelligence scores also mutually correlated with age and their associations were stronger, the relationship that exists between SSRT and intelligence could possibly be mediated by age. Therefore, we analyzed the components of variance in explaining VIQ, PIQ, and RCPM by employing the method used in a similar study by Michel and Anderson (2009), which involves calculating the differences of *R*^2^-values obtained from a series of regression analyses and determining the age-related and unique contribution of the predictor variables of interest to an outcome variable. Three separate analyses were carried out for VIQ, PIQ, and RCPM, in each case the predictors were age and SSRT. The variance inflation factor was 1.26, suggesting that multicollinearity was not violated. Model assumptions of linearity of relationships and normality and homoscedasticity of residuals were also met. The results of the regression analyses and components of variance calculation are shown in Tables 4, 5 and Figure 3.

**Table 4:** **Regression analyses examining the contribution of age and SSRT to VIQ, PIQ, and RCPM**.

	**Predictors**	**VIQ**	**PIQ**	**RCPM**
①	Age, SSRT	*R*^2^ = 0.333, *F*_(2, 147)_ = 36.62, *p* < 0.001	*R*^2^ = 0.310, *F*_(2, 147)_ = 33.08, *p* < 0.001	*R*^2^ = 0.157, *F*_(2, 147)_ = 13.74, *p* < 0.001
②	Age	*R*^2^ = 0.310, *F*_(1, 148)_ = 66.63, *p* < 0.001	*R*^2^ = 0.308, *F*_(1, 148)_ = 66.00, *p* < 0.001	*R*^2^ = 0.157, *F*_(1, 148)_ = 27.51, *p* < 0.001
③	SSRT	*R*^2^ = 0.148, *F*_(1, 148)_ = 25.70, *p* < 0.001	*R*^2^ = 0.085, *F*_(1, 148)_ = 13.69, *p* < 0.001	*R*^2^ = 0.041, *F*_(1, 148)_ = 6.38, *p* = 0.013

**Table 5:** **Components of variance in explaining VIQ, PIQ, and RCPM**.

	**Components**	**Derived from *R*^2^ by**	**VIQ**	**PIQ**	**RCPM**
④	Unique contribution of age	①–③	0.185 (=0.333–0.148)	0.225 (=0.310–0.085)	0.116 (=0.157–0.041)
⑤	Unique contribution of SSRT	①–②	0.023 (=0.333–0.310)	0.002 (=0.310–0.308)	0.000 (=0.157–0.157)
⑥	Age-related contribution of SSRT	①–④–⑤	0.125 (=0.333–0.185–0.023)	0.083 (=0.310–0.225–0.002)	0.041 (=0.157–0.116–0.000)

**Figure 4:**
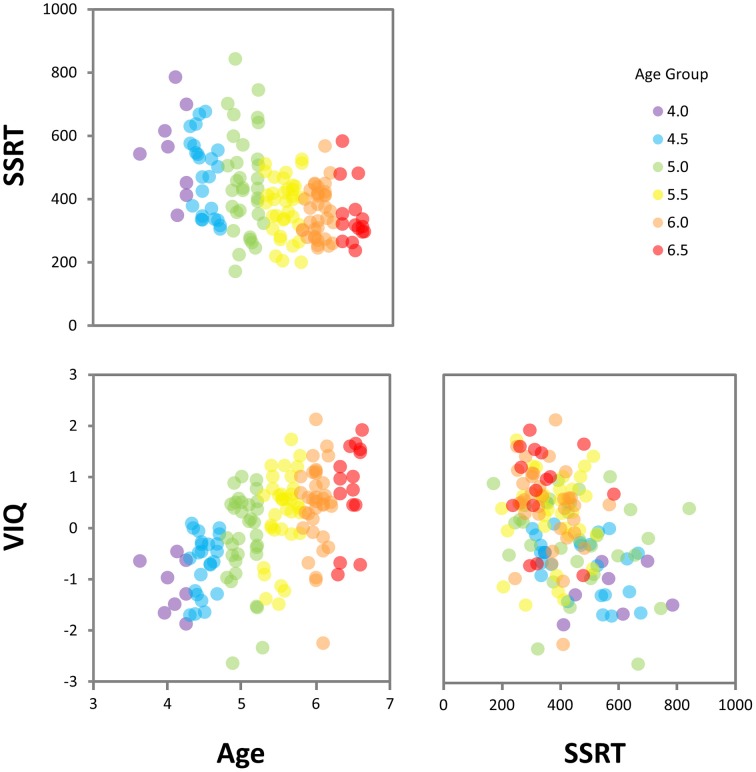
**Scatterplot matrix showing relationships between age, SSRT, and VIQ**. (SSRT, stop-signal reaction time; VIQ, Verbal IQ score).

Results of the regression analyses (see Table 4) showed that age and SSRT combined to explain 33% of the variance in VIQ. Notably, SSRT predicted unique variance in VIQ [β = −0.17, *t*_(147)_ = −2.21, *p* = 0.029] beyond that accounted for by age [β = 0.48, *t*_(147)_ = 6.38, *p* < 0.001]. As shown in the components of variance results (see Table 5), the age-related contribution of SSRT to VIQ was 13%, which was much larger than the unique contribution of SSRT (2%), indicating that SSRT predicted a considerably larger proportion of the age-related variance in VIQ than non-age-related variance. The relationships between age, SSRT, and VIQ are depicted in the scatterplot matrix in Figure 4.

Although the two fluid intelligence measures, PIQ and RCPM, were also predicted by both age and SSRT when entered together in the regression analyses (see Table 4), the contribution from SSRT was not significant [PIQ: β = −0.05, *t*_(147)_ = −0.65, *p* = 0.517; RCPM: β = −0.03, *t*_(147)_ = −0.36, *p* = 0.722]. The components of variance analyses (see Table 5) revealed that age uniquely contributed to most of the explained variance in PIQ and RCPM (23 and 12% of the variance, respectively). Whereas, the age-related contribution of SSRT to PIQ (8%) and RCPM (4%) was relatively low, its unique contribution was hardly noticeable. This indicates that the relationship that exists between SSRT and fluid intelligence in preschool children were already mediated by age.

## Discussion

The present study aimed to investigate the relationship between inhibitory control and intelligence by, firstly, charting the patterns and rates of development of response inhibition as indexed by SSRT and different types of IQ as measured on the WPPSI-R and RCPM during the preschool period, and secondly, by determining whether SSRT is differentially related to individual and age-related differences in children's intelligence.

We found that improvement with age in SSRT is in broad tandem with rises in VIQ between the ages of 4 and 6.5, but PIQ and RCPM showed rather different patterns of development. The fluid abilities assessed by PIQ and by RCPM not only began developing much later than did VIQ (at around 5–6 years of age) but also showed disparate developmental paths. The clearly separate developmental trajectories in early childhood intelligence provide further evidence that intelligence is not unitary and highlight the importance of studying how inhibitory control interacts with the dimensionality of intelligence during development.

The present study also found that the relationship between SSRT and intelligence was primarily age-related. Our components of variance analyses showed that the unique contribution of SSRT to VIQ, PIQ, and RCPM was exceedingly small compared to the age-related contribution of SSRT. In addition, of the three IQ measures, only the contribution from SSRT to VIQ was significant. Together, these results suggest that SSRT was associated with age-related changes in VIQ but not individual differences in any of the IQ scores. Put simply, children's inhibitory efficiency as indexed by SSRT is a contributing factor for the magnitude of the age differences in verbal intellectual functioning within the preschool period; however, when keeping age constant, it does not explain the differences in IQ between individual children. Rather than inhibitory control, Friedman et al. (2006) found that updating working memory correlates more highly with individual differences in intelligence. Gottfredson (1998) also suggests that genetics may contribute more to stable differences in intelligence among individuals than other factors.

With regard to age-related changes in intelligence, it is intriguing that performance on the stop-signal task, which is essentially a cognitive task that places a minimal load on verbal ability, is linked to the verbal aspects of intelligence in the context of development. This finding may seem counterintuitive because inhibition is traditionally thought to be related to fluid intelligence, which is involved in problem solving (e.g., Michel and Anderson, 2009). In fact, our data do not directly contradict this relationship because inhibitory control has been proposed to explain individual differences in fluid intelligence in conditions where both working memory and interference control are tapped (Kane and Engle, 2002; Engle and Kane, 2003; Gray et al., 2003; Burgess et al., 2011; Chuderski et al., 2012). In the case of the stop-signal task, it is largely knowledge-free and its demands on participants' working memory appear minimal, which may explain the lack of a relationship between SSRT and fluid intelligence. There might also be a possibility that because our results showed fluid abilities began developing much later than verbal IQ, their relationship to SSRT was not readily apparent during this age range.

The observed age-related relationship between inhibitory control and verbal ability is not manifestly inconsistent with the developmental literature (e.g., Vaughn et al., 1984; Michel and Anderson, 2009). It could possibly be argued that this relationship between the development of response inhibition and VIQ is attributable to their common underlying mechanisms which are mediated by frontal lobe functioning (Arbuckle and Gold, 1993). Nevertheless, the lack of a similar relationship between SSRT and fluid intelligence, which is also a cognitive ability subserved by the frontal lobes (Duncan et al., 2000; Gray et al., 2003), may render this explanation less satisfying.

One plausible explanation is that maturation of the inhibitory systems may be a factor underlying improvement in verbal intelligence. As Anderson (1992) argues, what changes in intellectual development is not the speed of processing but rather the content and organization of knowledge. With increasing age, a better developed capacity for inhibition not only improves suppression of task-irrelevant processing but also further enhances task-relevant processing, which enables one to better attend and respond selectively during learning (Davis and Anderson, 2001) and facilitates organization of such content and acquisition of higher order knowledge (see also Dempster, 1992; Craik and Bialystok, 2006). In this regard, selective attention and inhibitory control could be viewed as two complementary and allied functions.

Given that our data are correlational in nature and definitive causations cannot be assumed, this relationship between SSRT and VIQ could also be argued in the other direction. One alternative explanation might be that improvement in verbal abilities contributes to the development of inhibitory control. Self-regulatory behavior has been found to involve self-directed verbalization (Luria, 1961; Schunk, 1986; Barkley, 2001) and the use of language and symbolic representation (Baron and Gioia, 1998). Verbal ability is directly linked to efficiency of inhibitory control because both of them interact and evolve with development. However, this explanation would imply that efficiency of inhibitory control should be related to not only age-related changes but also individual differences in intelligence, because in this case the use of speech or language is directly linked to self-regulation. This, unfortunately, is incongruent with our findings.

## Conclusions

This study contributes to the understanding of the relationship between inhibitory control and intelligence in children of preschool years by showing that development of response inhibition is closely linked to improvement in verbal intelligence as a function of age. These results were obtained from a sizable sample and a psychometric measure that shows good validity and reliability as both an age measure and an individual difference measure of inhibitory control. One parsimonious view is that the processing of task-relevant and task-irrelevant information and responses is fundamental to intellectual functioning, and that these two functions are related in development perhaps because, as a child grows, more efficient intellectual functioning is partly dependent on the qualitative changes to accumulated knowledge that relies on a higher capacity for inhibition. To a large extent, these findings concur with Anderson's two-dimensional model of intelligence and development, but the present study also adds to the understanding of this conception by suggesting that it could be age changes in the verbal aspects of intelligence that are related to inhibitory control. Given the significance of age in explaining unique variance in PIQ and RCPM in our results and substantial research showing a physiological and genetic basis for fluid abilities, it seems plausible to argue that it is individual differences in fluid intelligence that are related to speed of cognitive processing.

Although the findings in this study were obtained from investigating stop-signal inhibition in preschool children, we speculate that its relationship to intellectual development would remain valid in other age groups and in other forms of inhibitory control (such as interference control) considering that they are equally concerned with an ability to cope with situations where task-relevant and task-irrelevant information or processes are in competition (Friedman and Miyake, 2004). It should be noted, however, that only one-third of the variability in VIQ was jointly explained by age and response inhibition as indexed by SSRT. Evidently, the remaining proportion of the variability in VIQ would be associated with factors not included in this study.

The above results should be interpreted in terms of the limitations of the present study. The results in our study were obtained from a Taiwanese sample, and there might possibly be cultural differences in performance on inhibition and intelligence measures (Shonkoff and Phillips, 2000; Sabbagh et al., 2006). The use of a cross-sectional design in investigating age-related changes may not reveal differences related to individual characteristics or birth cohorts. Future research may verify the direction of relationship between SSRT and VIQ by using a longitudinal design, further delineate the mechanisms involved in the development of inhibitory control and verbal intelligence, and expand the scope of the present study by exploring whether there could be other factors that may similarly affect the development of inhibitory control and intelligence.

The current study has broader implications for experimental design and for education. Firstly, the results from this study further highlight the importance of distinguishing between individual and age differences in intelligence in experimental studies, especially developmental studies. Secondly, if age changes in intelligence are related to the maturation of inhibitory systems, the provision of education to children should be tailored to their overall development as much as, if not more than, their levels of intelligence.

## Author contributions

Study design: CJ. Collection and assembly of data: YL, KL, WS. Data analysis and interpretation: HL, YL, KL, WS. Manuscript writing: HL, CJ.

### Conflict of interest statement

The authors declare that the research was conducted in the absence of any commercial or financial relationships that could be construed as a potential conflict of interest.

## Footnotes

^1^The remaining half of the items were reserved for a follow-up study as a post-test measurement.
